# Return to work after myocardial infarction: A systematic review

**DOI:** 10.34172/jcvtr.025.33351

**Published:** 2025-12-17

**Authors:** Tais Santana Barbosa, Natasha Cordeiro dos Santos, Millena Pereira Costa, Roque Aras Junior

**Affiliations:** ^1^Roberto Santos General Hospital, Bahia, Brazil; ^2^Federal University of Bahia, Bahia, Brazil

**Keywords:** Myocardial infarction, Return to work, Cohort study

## Abstract

The majority of people experiencing Myocardial Infarction are of working age, which may result in prolonged work disability. This study seeks to consolidate the available evidence regarding the return to work for individuals following a Myocardial Infarction, while also examining its correlation with disease severity, job engagement, and duration of hospitalization. This research is a systematic review. The databases utilized include MEDLINE, Lilacs, Scielo, and Web of Science, with keywords and synonyms sourced from the Health Sciences Descriptors (DeCS), Medical Subject Headings (MeSH), and Embase Subject Headings (Emtree). Data collection took place between November 2023 and June 2024. The studies’ quality was evaluated using the Quality Assessment Tool for Observational Cohort and Cross-Sectional Studies. The search yielded 4,695 articles, from which 12 cohort studies were selected for inclusion, encompassing a total of 83,702 participants. The rate of return to work fluctuated throughout the follow-up period, with a return rate of 21.5% to 41.7% after one month and between 76.9% and 92.7% after one year. Additionally, the studies reported on modifications in work roles, salary reductions, job dismissals, and the incidence of anxiety and depression. All cohort studies were deemed to be of good quality. The rate of returning to work following a Myocardial Infarction is notably high within one year and is associated with physical, psychological, and social factors, highlighting the need for mechanisms that facilitate this return as promptly as possible. However, further research is necessary, particularly involving diverse populations and distinguishing among different professional categories, to gather more extensive data.

## Introduction

 According to the World Health Organization (WHO), Cardiovascular Disease (CVD) is responsible for approximately 17.9 million deaths each year, positioning it as the foremost cause of mortality globally by 2030.^1^ Among its various manifestations, Myocardial Infarction (MI) stands out as a significant contributor to both illness and death worldwide, including in Brazil.^2^ From January 2012 to December 2021, Brazil recorded a staggering 1,103,858 hospital admissions attributed to Myocardial Infarction.^3^

 In this scenario, the demand for both clinical and surgical interventions, along with the complications arising from Myocardial Infarction and necessary lifestyle adjustments for patients, substantially influences their physical health, psychological well-being, financial stability, and social interactions. ^4^ It is recognized that beyond conventional outcomes such as mortality rates and readmission statistics, a crucial determinant of functional recovery and health-related quality of life (HRQoL) is the capacity to return to work. ^5^ Consequently, it becomes imperative to identify the facilitators and/or obstacles that may influence this return-to-work process.

 It is important to highlight that a majority of individuals who experience Myocardial Infarction are typically of working age, which can result in prolonged work disabilities.^6^ Although the 2023 guidelines from the European Society of Cardiology (ESC) emphasize that maintaining or regaining employment constitutes a vital aspect of recovery post-Myocardial Infarction, ^7^ medical professionals frequently prescribe sick leave as part of rehabilitation strategies for these patients. ^8^

 Within this framework, those patients who do not resume work within six months following a Myocardial Infarction are statistically less likely to achieve reintegration into the workforce in subsequent years. ^9^ Therefore, collating information on the return-to-work experiences of Myocardial Infarction patients—alongside its correlation with disease severity, occupational roles, and duration of hospitalization—can aid in formulating tailored interventions for these individuals while simultaneously mitigating healthcare expenditures. This study aims to consolidate existing research regarding the return-to-work dynamics for individuals recovering from Myocardial Infarction and explore its association with disease severity, job engagement, and length of hospital stay.

## Methods

 This systematic review was carried out following the PRISMA (Preferred Reporting Items for Systematic Reviews and Meta-Analyses) guidelines established by Moher et al.^10^ The research utilized several databases, including MEDLINE (Medical Literature Analysis and Retrieval System Online), Lilacs (Latin American and Caribbean Health Sciences Literature), SciELO (Scientific Electronic Library Online), and Web of Science. Data collection occurred between November 2023 and June 2024.

 The study was designed using the PICOS framework, which stands for Population (individuals who experienced Myocardial Infarction), Intervention or Exposure (hospitalization due to Myocardial Infarction), Comparison (severity, occupational activity, and duration of hospitalization), Outcomes (return to work), and Study design (cohort study). Keywords and their synonyms were selected based on the respective databases, referencing the Health Sciences Descriptors (DeCS), Medical Subject Headings (MeSH), and Embase Subject Headings (Emtree). Boolean operators “AND” and “OR” were utilized as detailed in Table 1. The search strategy focused on terms appearing in article titles, subject headings, and abstracts.

**Table 1:** Keywords used in the electronic search combined with the boolean operators “AND” and “OR”

	**Keywords**	**Synonyms**
DECS	Myocardial Infarction	Myocardial Infarct, Myocardial Infarctions, Myocardial Infarcts
	Return to Work	Back to Work, Back-to-Work, Return-to-Work
	Cohort Study	Cohort Analysis
MESH	Myocardial Infarction	Myocardial Infarctions, Myocardial Infarctions, Myocardial Infarcts
	Return to Work	Back to Work
	Cohort Study	Cohort Studies
EMTREE	Myocardial Infarction	Myocardial Infarctions, Myocardial Infarctions
	Return to Work	Back to Work
	Cohort Study	Cohort Studies

 Cohort studies assessing the return to work for individuals who experienced a Myocardial Infarction were included in this review, without any restrictions based on language. Studies that also addressed other conditions within the same cohort, such as Heart Failure and Rheumatic Heart Disease, were excluded. Relevant articles identified through database searches underwent a systematic selection process consisting of three stages: initial title screening, followed by abstract evaluation, and finally a comprehensive reading of the full text.

 Subsequently, an exploratory review of all selected materials was conducted, leading to a focused and analytical reading of the most pertinent sections. The identification of methodological features and data extraction from these articles was performed by two independent reviewers. In instances of disagreement, both reviewers revisited the entire article for reevaluation. If discrepancies remained unresolved, a third independent reviewer was brought in to provide further insight.

 The quality assessment of the studies included in this review utilized the Quality Assessment Tool for Observational Cohort and Cross-Sectional Studies provided by the National Heart, Lung, and Blood Institute (NHLBI). Information from the articles was systematically organized into tables detailing key research attributes such as first author, publication year, study type, database utilized, classification of Myocardial Infarction, inclusion criteria, sample size, along with outcomes studied and their principal findings and conclusions.

 The protocol outlining the various stages involved in constructing this systematic review has been submitted to the International Prospective Register of Systematic Reviews (PROSPERO), under registration number CRD42023464316.

## Results

**Figure 1:**
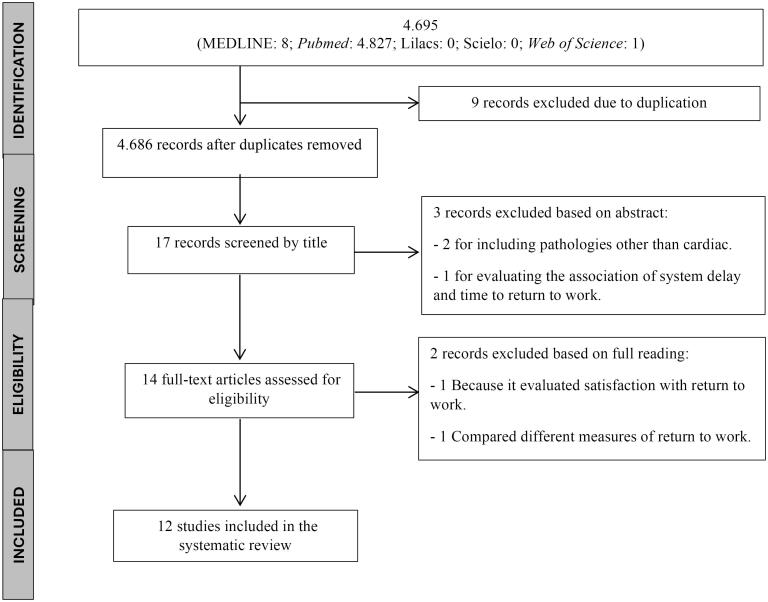
Flow diagram of the study search and identification process, according to the PRISMA methodology

 The search of the database yielded a total of 4,695 articles (MEDLINE: 8; PubMed: 4,686; Lilacs: 0; Scielo: 0; Web of Science: 1). After eliminating duplicates, 4,686 records remained. From these, 17 articles were chosen through title screening. Three articles were excluded due to their abstracts failing to satisfy the selection criteria, which left 14 articles for comprehensive review. Of those reviewed, 2 did not fulfill all inclusion requirements, leading to a final count of 12 articles, as illustrated in Figure 1.

 The twelve articles were meticulously reviewed and categorized, resulting in the creation of three tables that encapsulate pertinent research details. These tables include key attributes such as the first author’s name, publication year, study design, country of origin, classification of Myocardial Infarction, sample size, and criteria for inclusion (refer to Table 2). Table 3 summarizes the outcomes investigated, principal findings, and overall conclusions derived from the studies. Additionally, Table 4 provides information regarding disease characterization, duration of hospital stay, time required for return to work, and associated comorbidities. All studies included in this review were cohort studies, comprising nine prospective studies^11-19^ and three retrospective ones.^20-22^ The geographical origins of the research spanned Europe,^12,14,16-18,20,21^ Asia,^11,13,22^ Oceania,^19^ and North America.^15^ The inclusion criteria prominently featured the diagnosis of Myocardial Infarction,^11,12,14,15,18,20-22^ age ( > 18 ^15,22^, 18-63 ^20^, ≥ 55 for women and ≥ 60 for men,^13^ < 60^16^ > 18-60,^14^ 18-64,^17^ 30-65,^21^ ≤ 75^19^), and employment status at the time of or prior to the event. Collectively, these studies involved a total of 83,702 participants, with sample sizes ranging from as few as 102^12^ to as many as 22,394 ^21^ volunteers. A predominance of male participants was noted, with average ages at both minimum (48.65 years)^22^ and maximum (64.35 years)^11^ extremes.

**Table 2:** Main characteristics of the articles included in the Systematic Review (n = 12)

**Author, year**	**Study Type**	**Country**	**Type of MI**	**Sample**	**Inclusion Criteria**
Sun et al, 2022^11^	Longitudinal prospective cohort study.	South Korea	STEMI (58,1%)	- Total of 136 participants; - Average age: 64.35 years, with ages spanning from 21 to 86 years; - Male participants constituted 73.5%; - Married individuals made up 87.5%; - Over half possessed a high school diploma or higher education (58.0%); - At the commencement of the study, less than half were employed (41.2%), and a significant majority rated their financial condition as either "adequate" or "poor" (86.8%).	- Received a diagnosis of myocardial infarction (MI) from a cardiologist; - Possesses sufficient proficiency in the Korean language to accurately complete the study questionnaires; - Has been a resident of South Korea; - Exhibited capacity to comprehend the study requirements and provide written informed consent; - Recognized various forms of paid work, including regular employment, occasional jobs, and self-employment, as valid employment status; - The analysis also included participants who were unemployed prior to the MI, acknowledging that they might still have the potential to seek employment following recovery from the MI.
Stendardo et al, 2018^12^	Prospective cohort study.	Italy	NSTEMI 64 (62,75%).	- A total of 102 patients were included in the study. - The median age of participants was 56 years (IQR 50-60). - The majority of the cohort consisted of male patients (88.24%) and those who were employed (68.63%). - Among them, 76.47% identified as smokers or ex-smokers, and 86.46% (83 individuals) resided with a partner. - Participants were engaged in either office/coordination roles or manual labor, comprising 47.06% and 52.94%, respectively; notably, only 12 subjects (12.76%) held a university degree.	- Diagnosis of myocardial infarction based on the criteria established by the European Cardiac Society; - Individuals who received treatment through percutaneous coronary intervention and those who were employed at the time of the incident.
Jiang et al, 2018^13^	Prospective cohort study.	China	- STEMI 103 (6,6%) patients; - 627 (40,0%) had inferior wall MI; - 315 (20,1%) had anterior wall MI.	- A total of 1,566 patients participated from 53 hospitals. - The average age was 52.2 years (SD = 9.7), with 169 individuals (10.8%) aged 65 years or older. - Among the participants, 130 individuals (8.3%) were identified as women. - Educational attainment showed that 267 individuals (17.0%) held a bachelor's degree or higher, while 550 participants (35.1%) were employed in agricultural sectors. - Notably, 1,462 patients (93.4%) were married.	- Individuals who indicated they were working (either part-time or full-time) during their hospitalization for a myocardial infarction (MI), consented to participate in follow-up interviews, were discharged in stable condition, did not transfer to another intensive care facility, and successfully completed the 12-month interview. - Those patients who did not resume work following their MI may have chosen to retire, which excludes 96 employed individuals who had reached retirement age (typically defined as ≥ 55 years for women and ≥ 60 years for men) by the time of the 12-month interview.
Butt et al, 2018^14^	Prospective cohort study.	Denmark	Not specified	- 6,031 Individuals - The median age was 55 years, with a male representation of 88.9%. - A significant number of participants had vocational training and basic education, accounting for 2,675 and 1,730 individuals, respectively. - Younger patients (ages 18-45) were more frequently subjected to emergency and urgent surgical procedures. They exhibited a lower male ratio, had fewer comorbid conditions, but experienced higher rates of readmission during the first year post-discharge compared to their older counterparts.	- Identified all Danish individuals who underwent their initial isolated coronary artery bypass grafting (CABG). - Included exclusively working-age patients aged between 18 and 60 years, who were actively part of the labor force 30 days prior to admission and were discharged alive following myocardial revascularization. - Patients were categorized as part of the workforce if they were employed, unemployed but capable of work (i.e., not on paid sick leave, disability pension, or early retirement), or receiving state educational grants, paid maternity leave, or other forms of leave.
Warraich et al, 2018^15^	Longitudinal observational study.	United States	STEMI 2.417 (57%)	- Total Participants: 9,319 - Average Age: 55.8 years (SD 9.2), with 27.3% identifying as female - Employment Status at Onset of MI: Over half of the participants (n = 4,730, representing 51%) were employed - Demographics of Non-Working Patients: Individuals not engaged in work at the study's commencement tended to be older, predominantly Black, female, single, and generally lacked a high school diploma. Additionally, they exhibited a higher prevalence of medical comorbidities compared to their working counterparts - Retirement Among Non-Workers: A significant majority of those not employed at the study's start were retired (n = 3,355, accounting for 73%) - Characteristics of Working Patients: Those who maintained employment during the initial phase were more likely to engage in smoking and exhibited a higher incidence of STEMI or experienced cardiac arrest during hospitalization related to MI.	- Patients were eligible for inclusion in the registry if they were 18 years of age or older, diagnosed with either STEMI or NSTEMI, received treatment via PCI along with a P2Y12 inhibitor, were not participating in any other research studies, and could provide written consent for ongoing telephone follow-up and data gathering.
Babić et al, 2015^16^	Prospective, single-center, open study.	Croatia	STEMI	- The average age of the 145 patients examined was 53.17 years, with a standard deviation of ± 7.29. - A total of 101 patients, representing 69.7%, had completed their high school education. - Rehabilitation was carried out for 48.3% of the patients. - Employment distribution revealed that 54.4% worked in government sectors, while 16.6% were employed by private firms with fewer than 100 employees, 11.7% in larger private companies employing over 100 individuals, and 14.5% operated their own businesses; additionally, 2.1% were engaged in a mix of these employment types.	- Participants were required to be under the age of 60 and currently employed. - The duration of the follow-up was established at two years. - Diagnosis of STEMI was conducted in accordance with the criteria set by the European Cardiac Society at the time of the study.
Osler et al, 2014^17^	Cohort study.	Denmark	Most people who RTW had unstable angina (55.1%), 5,773 had STEMI, 4,136 NSTEMI, and 6,542 unspecified MI.	- 21,926 Participants - The predominant age group among respondents was 50 to 59 years, comprising 11,010 individuals, with 59.7% currently on medical leave. - Among the employed participants, females represented 38%, while males accounted for 37%. - A significant majority resided with a partner (14,744 individuals) and possessed a high school diploma (10,071 individuals). - There were 12,927 individuals categorized as entry-level employees.	- The National Patient Registry classified all initial hospitalizations for Acute Coronary Syndrome (ACS) as unstable angina, ST-Elevation Myocardial Infarction (STEMI), Non-ST-Elevation Myocardial Infarction (NSTEMI), and myocardial infarction (MI). - Participants who were either younger than 18 or older than 64 years, those who retired due to disability or opted for early pension prior to diagnosis, and individuals who passed away within the first 30 days post-diagnosis were not included in the study.
de Jonge et al, 2014^18^	Prospective cohort study	Netherlands	Unspecified	- Total Participants: 186 - Average Age of Participants Returning to Work (RTW): At 3 months, the mean age was 50.9 years (SD = 8.2), and at 12 months, it was 49.7 years (SD = 6.7). - Gender Distribution: A significant proportion were male, with RTW rates of 78 (90.7%) at 3 months and 122 (91.7%) at 12 months. - Education Level: The majority possessed lower to medium-level secondary education qualifications. - Job Type for Participants RTW: Among those who returned to work after both time frames, a considerable number held sedentary positions: 39 (45.3%) at 3 months and 55 (41.4%) at 12 months. - Workload Classification: For participants returning to work at both intervals, the workload was predominantly categorized as light: with counts of 54 (62.8%) and 70 (52.6%) respectively at the two time points.	- To qualify, patients needed to satisfy a minimum of two criteria: (1) experiencing chest pain for at least 20 minutes, (2) having elevated levels of enzymes such as creatine phosphokinase and creatine phosphokinase-MB, and (3) demonstrating new pathological Q waves on the electrocardiogram across at least two leads. - Furthermore, an additional requirement for inclusion was that patients must have been employed in the year preceding their myocardial infarction (MI).
Worcester et al, 2014^19^	Prospective, longitudinal, observational cohort study	Australia	Unspecified	- 401 Patients - In comparison to clinical patients, those undergoing surgery had an average age of 57.77 years (SD = 7.89), contrasted with a mean age of 52.1 years (SD = 8.12); *P* < 0.00. - Surgical patients were more frequently homeowners (*P* < 0.001), possessed private health insurance (*P* < 0.001), reported a history of myocardial infarction (MI) (*P* < 0.001), experienced prior angina episodes (*P* < 0.001), and exhibited signs of depression (*P* = 0.006). - Furthermore, surgical patients showed a significantly lower likelihood of having impaired left ventricular ejection fraction (LVEF) (*P* = 0.009), working in manual occupations (*P* = 0.002), or being current smokers (*P* < 0.001).	- The criteria for inclusion consisted of being employed within two months following hospital admission and being 75 years old or younger.
Lauridsen et al, 2022^20^	Danish national cohort study.	Denmark	STEMI with CS	- Total participants: 19,799, comprising 19,146 individuals without CS and 653 with CS (3%); - The median age for both groups was comparable at 53 years (IQR 47-58), with similar demographics in terms of income, education, and health burden; - A higher prevalence of male patients was noted among those with CS.	- Individuals were identified through the Danish National Patient Register, specifically those with their initial hospitalization due to STEMI. The diagnosis of CS was established either through a corresponding diagnosis code for CS or by any administration of vasoactive medications during the myocardial infarction hospitalization. - The study focused exclusively on working-age individuals (ages 18–63) who were employed prior to their hospitalization and remained alive post-discharge. - Participants were categorized as workers if they were currently employed, unemployed yet capable of working, or receiving state educational support, maternity leave benefits, or other forms of leave.
Smedegaard et al, 2017^21^	Nationwide retrospective cohort study.	Denmark	Not specified	- Among the patients, 22,394 individuals (56.9%) were employed prior to their first myocardial infarction (MI). - The study group comprised 18,120 men (80.9%), with a median age of 55 years (interquartile range: 49-59). - The predominant educational attainment was vocational training at 45.1%, followed by basic education at 30.4%.	- Individuals between the ages of 30 and 65 who received a diagnosis of myocardial infarction (MI) upon their initial primary discharge were considered for inclusion. - Participants must have been employed for a minimum of three consecutive weeks within the five-week timeframe leading up to their MI admission date. - Inclusion criteria extended to patients who were admitted within 30 days prior to the cutoff date.
Mohd Mustafah et al, 2017^22^	Retrospective cohort study.	Malaysia	46 (63.9%) had STEMI and 18 (25.0%) NSTEMI among RTW.	- 112 Individuals - The participants who resumed work had an average age of 48.65 years ( ± 7.78), with a predominant male representation of 73 individuals (98.6%). - A significant portion, totaling 42 participants (56.7%), possessed a secondary education level. - In terms of employment sectors, 46 individuals (62.2%) were employed in the private sector, with 41 of them (55.4%) classified within skilled occupations, followed by 20 participants (27.0%) working as business professionals.	- All participants were patients in rehabilitation following a cardiac incident (myocardial infarction, percutaneous coronary intervention, or coronary artery bypass grafting) and were currently on medical leave. - The criteria for inclusion required that participants be at least 18 years old, have been employed prior to the cardiac event, and possess the ability to communicate.

Legend: MI – Myocardial Infarction; STEMI - ST-Elevation Myocardial Infarction; CS - Cardiogenic Shock; IQR – Interquartile Range; NSTEMI - Non-ST-Elevation Myocardial Infarction; SD – Standard Deviation; PCI - Percutaneous Coronary Intervention; RTW – Return to Work; CABG - Coronary Artery Bypass Grafting.

**Table 3:** Main outcomes of the articles included in the Systematic Review (n = 12)

**Author, year**	**Outcomes**	**Main results**	**Conclusions**
Sun et al,2022^11^	- The percentage of myocardial infarction patients in South Korea who resumed work three months post-discharge. - Various demographic, behavioral, and clinical factors were identified as predictors influencing these patients' return to the workforce.	- Of the 136 participants, 56 individuals (41.2%) were employed at the time of their myocardial infarction (MI), while 80 individuals (58.8%) were not in employment. At the three-month follow-up, 87.5% (n = 49) of those who had jobs at the beginning of the study successfully returned to work. - Participants who did not re-enter the workforce within three months exhibited a higher likelihood of being female, possessing lower educational qualifications, currently smoking and consuming alcohol, having diabetes and additional comorbid conditions, as well as experiencing readmission to a hospital post-discharge (all *P* < 0.05). - Additionally, older participants, those with diminished social support networks, and individuals displaying more pronounced symptoms of anxiety and depression were found to be less likely to resume work three months following their discharge.	The primary findings of this research indicate that most employed patients who undergo a myocardial infarction (MI) are capable of resuming their professional duties within three months following their discharge.
Stendardo et al, 2018^12^	- Examine the influence of physical, psychological, sociodemographic, and occupational variables on the likelihood of returning to work within one year following a myocardial infarction (MI) in a uniform group of patients who have undergone coronary angioplasty and did not experience a postoperative MI.	- At the one-month follow-up appointment, 60 individuals (59.80%) indicated they had not experienced any cardiac symptoms since being discharged. The estimated median MET values reflecting patient exercise capacity were calculated at 6.03 (IQR 5.50–6.53). Scores from both HADS questionnaires measuring anxiety and depression, along with spirometric parameters (FEV1, FVC, and FEV1/FVC), fell within normal reference ranges. - On day 30, 78.5% of participants had not resumed work; this figure decreased to 40.8% by day 60, and by day 365, only 7.3% remained out of work. - The median duration of absence from work was recorded at 44 days (IQR 33–88). - Key qualitative variables identified as significant for facilitating an earlier return to work included educational attainment (*P* = 0.026), self-employment status (*P* = 0.0005), and the professional classification associated with office or coordination tasks (*P* = 0.020). - Multivariate analysis supports that possessing a university degree, being self-employed, and maintaining a lower HADS-D score are associated with a greater likelihood of returning to work sooner.	The most significant factors influencing a return to work within one year following discharge from a myocardial infarction treated with percutaneous coronary intervention include being self-employed, possessing a higher level of education, and maintaining a positive emotional state.
Jiang et al, 2018^13^	- Return to work refers to resuming either full-time or part-time employment within a 12-month period following discharge from the initial hospitalization due to myocardial infarction (MI).	- A total of 875 patients (55.9%) resumed work within 12 months following hospitalization for a myocardial infarction (MI). Among the 691 patients who did not return to work, 287 (41.5%) were either unable or opted not to work due to their MI, 131 (19.0%) retired early as a result of the condition, 44 (6.4%) became full-time homemakers or were unemployed, while 229 (33.1%) did not provide a reason for their absence from the workforce. - In comparison to those who returned to work within the designated time frame, individuals who did not return tended to be older and had a higher likelihood of being female, employed in agricultural jobs at the time of their initial MI, suffering from hypertension, and experiencing significant adverse events such as stroke, atrial fibrillation, and angina during their hospital stay. - Factors that correlated with an increased probability of returning to work included possessing a college degree (RR, 1.30; 95% CI, 1.02-1.56), having a history of type 2 diabetes (RR, 1.24; 95% CI, 1.02-1.44), and experiencing an anterior wall MI (RR, 1.22; 95% CI, 1.01-1.41).	Among employed individuals hospitalized due to myocardial infarction (MI) in China, almost 50% did not resume their jobs within a year. Factors such as being female, having a history of smoking, and experiencing negative incidents during hospitalization were associated with a decreased likelihood of returning to work post-MI.
Butt et al, 2018^14^	- The main objective was to assess the return to employment, which was anticipated to occur within a timeframe ranging from 5 weeks to one year following coronary artery bypass grafting (CABG). - Additionally, the duration until individuals resumed work was evaluated, specifically defined as a minimum of 3 consecutive weeks at any point within a 2-year follow-up window. - The assessment also included the sustained connection to the workforce throughout the 2-year follow-up period.	- One year post-discharge, 4,827 patients (80.0%) re-entered the workforce, while 614 patients (10.2%) were on paid medical leave. Additionally, 63 patients (1.0%) required support due to diminished work capacity, 204 patients (3.4%) received disability pensions, 250 patients (4.1%) opted for early retirement, 57 patients (0.9%) had passed away, and 16 patients (0.3%) had emigrated. - The rate of workforce reintegration at the one-year follow-up varied between 75.6% and 84.2%, with the lowest rates observed in older age groups. - At discharge, 965 patients (16.0%) were noted to have comorbidities. - Factors enhancing the probability of returning to work included being younger, male, possessing a higher level of education, and having a greater income. - Over a two-year follow-up period, 5,524 patients (91.6%) returned to employment; among these individuals, 83.6% resumed work within six months following CABG discharge. Of those who returned to work for at least three consecutive weeks, 37.2% disengaged from their jobs within two years without taking medical leave. For those who accepted medical leave ranging from three to twelve weeks, the rates of disengagement dropped to 31.2% and 25.0%, respectively.	Working-age individuals who were employed in the month preceding their coronary artery bypass graft (CABG) surgery demonstrated a notable trend in workforce reintegration. A year following their discharge, approximately 80% of these patients successfully resumed their professional roles, accompanied by a low mortality rate.
Warraich et al, 2018^15^	- Examination of the occurrence of negative employment shifts from baseline to one year following myocardial infarction in a national cohort study conducted in the United States. - Analyzing patient-reported levels of depression, quality of life, adherence to medication regimens, and financial challenges related to acquiring medications among individuals experiencing adverse employment changes.	- Among patients who were employed at the start of the study, 10% (n = 492) experienced a negative shift in their employment status one year later. Of these, 7% (n = 349) reported being unemployed, while 3% (n = 143) indicated they were working fewer hours. Only 27 individuals who faced an adverse employment change cited retirement as the reason; notably, 172 patients (representing 49% of those who became unemployed) reported losing their jobs involuntarily due to circumstances such as termination or health-related issues. - Individuals who underwent an unfavorable change in employment following myocardial infarction (MI) tended to be more likely female, had a higher prevalence of diabetes and hypertension, utilized tobacco products, and were less frequently recipients of drug-eluting stents compared to those who maintained their previous employment status. - The most significant predictor of adverse employment changes within one year post-discharge was the frequency of readmissions during that same period, with an odds ratio of 1.20 (95% CI 1.09–1.32) for each event. - After one year, patients experiencing adverse changes in their employment were more likely to present with PHQ2 scores exceeding 3, indicating potential depression, alongside lower EQ5D VAS scores reflecting diminished quality of life. These patterns remained evident in both unadjusted and adjusted analyses.	Patients facing unfavorable changes in their employment status are more susceptible to depression, experience a diminished quality of life, and encounter increased financial burdens related to medication expenses when compared to those who remain employed. The most significant predictors of adverse employment shifts are unanticipated readmissions and bleeding events following a myocardial infarction, highlighting the critical need to mitigate the occurrence of these negative outcomes.
Babić et al, 2015^16^	- Examine various factors related to returning to work, socioeconomic issues, and overall quality of life among 145 employed patients under the age of 60 who have experienced STEMI and received treatment through primary PCI.	- The average duration of sick leave following a STEMI event was 125.83 days, with a standard deviation of 125.04 days. - Post-STEMI, 3.4% of patients left the workforce, while 31.7% opted for retirement. Overall, 17.9% of individuals reported a notable decrease in their salary compared to their earnings prior to the coronary incident. - Regarding quality of life assessments post-STEMI, 29.7% of patients felt it remained unchanged from before the event, while 40.7% perceived it as worse and another 29.7% viewed it as improved. - The correlation between salary levels before and after STEMI and self-reported quality of life changes was linked to the length of sick leave taken. - Age was found to have a significant correlation with the likelihood of permanent cessation from work. - Patients who experienced longer periods of sick leave typically had lower income levels both prior to and following the myocardial infarction (MI), which correlated with poorer quality of life assessments in this group. - A multivariate regression analysis indicated that alongside age (*P* < 0.001, odds ratio [OR] = 1.5), factors such as hyperlipoproteinemia (*P* < 0.05, OR = 0.32) and lower educational attainment (*P* < 0.05, OR = 0.04) were significantly associated with increased rates of discharge from work and retirement after experiencing STEMI.	Individualized cardiac rehabilitation must be prioritized and organized by the assigned team right after the acute phase of a myocardial infarction (MI). Insufficient health policies and delays in initiating cardiac rehabilitation post-MI can result in extended hospital stays, increased sick leave, and a diminished quality of life following the event, even when optimal treatment is administered during the acute stage of the condition.
Osler et al, 2014^17^	- To investigate how factors such as patient gender, comorbidity, and socioeconomic status influence later labor market participation (including categories like employed, unemployed, on sick leave, and those opting for disability or voluntary early retirement) within a group of working patients in Denmark.	- Among the 21,926 patients studied, 37% were employed 30 days post their initial admission for an Acute Coronary Syndrome (ACS) diagnosis. In contrast, 55% were on medical leave, and 8% were unemployed. - Employment rates were notably higher among male patients, younger individuals, those with higher education levels, those living with a partner, and those who experienced less severe or uncertain health events (such as being diagnosed with unstable angina without undergoing invasive treatment). Additionally, patients without comorbidities and those who were employed prior to their ACS diagnosis showed increased employment likelihood. - Of the participants who retained employment during the follow-up period, 34% experienced one or more instances of medical leave. Furthermore, 30% transitioned into disability or opted for voluntary early retirement following episodes of medical leave and/or unemployment. - Five years post-ACS diagnosis, a substantial 88% remained engaged in the labor market. Within this group, 65% held jobs, while 19% were unemployed and 16% were on medical leave. Most individuals exiting the labor market prior to age 64 received either disability pensions or chose voluntary early retirement. - Throughout the follow-up period, a total of 43% permanently exited the workforce. The average duration from 30 days post-diagnosis to retirement, death, or censoring was calculated at 4.1 years (with a median of 3.7 years; ranging from a minimum of 0.0 to a maximum of 10.2 years).	More than 50% of patients experiencing their first acute coronary syndrome (ACS) event either stay at work or return shortly afterward. However, certain groups—including women, those from socially disadvantaged backgrounds, individuals facing more severe cardiac incidents, and those with additional health conditions—exhibit lower rates of returning to work when other clinical variables are taken into consideration. It is essential to factor in these aspects during both physical recovery and social rehabilitation.
de Jonge et al, 2014^18^	- We analyzed the impact of depressive and anxiety disorders, identified through a diagnostic interview, on the rates of returning to work one year following a myocardial infarction (MI).	- After three months post-myocardial infarction (MI), 86 patients, accounting for 46.2%, resumed work, while 100 did not. - At the twelve-month mark, 133 individuals (76.9%) returned to their jobs, with 40 remaining out of work. - When comparing those who returned to work three months after MI with those who did not, it was found that the former group had notably higher educational attainment, a greater likelihood of holding sedentary positions, and were more frequently employed in less physically demanding roles. By twelve months following MI, individuals who went back to work tended to be younger, exhibited lower incidences of left ventricular ejection fraction (LVEF) below 40%, presented higher Killip class ratings, and were more prone to have experienced anterior MI than their counterparts who did not return to work. - Among the cohort of 160 patients, 31 were diagnosed with a major depressive episode according to ICD-10 criteria (19.4%), while 19 met the criteria for an anxiety disorder (11.9%). The occurrence of a major depressive episode did not serve as a predictor for non-return to work at twelve months post-MI [odds ratio (OR) 2.86; confidence interval (CI): 1.24–6.58; *P* = 0.013]. - A diagnosis under ICD-10 for any anxiety disorder within the initial three months following MI showed a nearly significant correlation with an increased risk of not returning to work (OR 2.58; CI: 0.96–6.97; *P* = 0.062).	The occurrence of a depressive episode was linked to a heightened risk of not resuming work among patients with myocardial infarction (MI). The relationship between anxiety and the likelihood of failing to return to work may, in some respects, be attributed to the co-occurrence of depression. Future research could explore the potential for mitigating the impact of depression through appropriate therapeutic interventions.
Worcester et al, 2014^19^	- To examine the employment re-entry rate within the first year following ACS or CABG procedures. - To record the employment status at the one-year mark. - To determine factors that influence the likelihood of not returning to work. - To uncover factors associated with a postponed return to work.	- RTW data were collected for a total of 378 patients. Among these, 343 individuals (90.7%) successfully returned to work, with 309 of them (91.1%) doing so within a four-month period. Conversely, 35 patients (9.3%) did not return to their jobs. At the one-year mark, 302 patients (79.9%) remained employed, while 32 (8.5%) were unemployed and 20 (5.3%) had retired. - The average duration until return to work was calculated at 8.62 weeks (SD = 7.66). Specifically, ACS patients had an average return time of 7.89 weeks (SD = 9.29), whereas CABG patients averaged 9.30 weeks (SD = 5.66). - Factors significantly associated with the lack of return to work included reporting fewer working hours prior to admission, experiencing financial stress, lacking a clear plan or feeling uncertain about resuming work, having significant comorbid conditions beyond diabetes mellitus, and holding a negative view of one's health. - No notable differences were observed between clinical and surgical patient groups regarding the proportion that returned to work, with rates at 91.2% for clinical and 90.3% for surgical patients. - There were observable trends indicating that jobs requiring physical activity and CABG patients who underwent concurrent valve surgery were associated with lower rates of return to work. - Key predictors for returning to work included engaging in manual labor or physically demanding occupations, experiencing job dissatisfaction, exhibiting depressive symptoms, lacking a supportive confidant, and maintaining a negative outlook on the prospect of returning to work. - Participation in cardiac rehabilitation was identified as a factor contributing to delayed return to work.	A substantial percentage of individuals who were employed prior to experiencing an acute cardiac incident tend to resume their jobs within a year. The inability to validate predictors of work return identified in previous studies can be attributed, in part, to the exceptionally high rate of employment reinstatement observed in our research, which took place during a period of low unemployment. Nonetheless, there exists a small yet notable group of patients who either did not go back to work, exited the workforce prematurely after initially returning, or were subsequently lost to follow-up.
Lauridsen et al, 2022^20^	- The relationship between myocardial infarction (MI) and chronic stress (CS) in the context of returning to work, job retention, and the various factors influencing this return.	- Among patients without cardiac surgery (CS), 83% were able to return to work within one year. Conversely, only 52% of patients who underwent CS managed to resume work. - The cumulative incidence of returning to work at six months, while accounting for the competing risk of mortality, was found to be 83% in the non-CS group, which rose to 91% by the one-year mark. In contrast, merely 53% of patients with CS returned to work after six months, and this figure increased to 67% after one year. - Patients carrying a greater burden of comorbidities demonstrated a reduced likelihood of returning to work, irrespective of their CS status. - Analysis using a multiple logistic regression model indicated that those who had undergone CS faced a diminished probability of re-entering the workforce when compared with their non-CS counterparts. - Male patients exhibited an enhanced likelihood of returning to work, with an odds ratio (OR) of 1.83 (95% confidence interval [CI]: 1.15–2.92) relative to female patients. - Additionally, individuals who experienced out-of-hospital cardiac arrest (OHCA) showed a higher chance of resuming work, reflected by an OR of 1.55 (95% CI: 1.00–2.40).	Approximately 80% of individuals without cardiac surgery (CS) resumed employment, whereas only 50% of those who underwent CS were back at work after one year. Nonetheless, once employed, both groups demonstrated comparable rates of secondary disengagement from work. For patients with CS, being male and surviving an out-of-hospital cardiac arrest (OHCA) were positively associated with a return to work, while extended hospital stays and anoxic brain injuries negatively impacted this outcome.
Smedegaard et al, 2017^21^	- Resuming employment within one year following the initial hospitalization for a myocardial infarction and the cessation of work after re-entering the workforce.	- Within a month following myocardial infarction (MI), 9,329 patients (41.7% [95% CI, 41.0%–42.3%]) resumed work. This figure rose significantly to 20,415 individuals (91.1% [95% CI, 90.7%–91.5%]) after one year, while there were 92 reported fatalities (0.4% [95% CI, 0.3%–0.5%]). When assessing employment after three months, the number of patients who returned to work totaled 19,369 (86.4% [95% CI, 86.0%–86.9%]). - Identified risk factors contributing to the likelihood of not returning to work included female gender (OR 1.43 [95% CI, 1.27–1.60]), heart failure (OR 2.36 [95% CI, 2.07–2.69]), arrhythmia (OR 1.67 [95% CI, 1.44–1.94]), cerebrovascular disease (OR 1.83 [95% CI, 1.44–2.31]), and chronic kidney disease (OR 1.46 [95% CI, 1.03–2.04]). - Factors that positively influenced the return to work encompassed a higher educational attainment and increased income levels for men; however, these financial advantages did not extend similarly to women. - Among the cohort of patients who re-entered the workforce—20,415 in total—5,169 individuals (25.3%) experienced termination from their jobs within a year post-return; of these former employees, 4,938 (24.2% [95% CI, 23.6%-24.8%) received social assistance benefits while there were also reports of 231 deaths (1.1 % [95 % CI, 1.0 % -1.3 % ]). - Additional predictors associated with job detachment included heart failure, diabetes mellitus (DM), arrhythmia, cerebrovascular disease, chronic kidney disease and depression.	91% of individuals resumed employment; however, within the following year, 24% of these patients lost their jobs, reflecting a detachment rate three times greater than that of the general population. Key factors influencing this employment loss included being younger (ages 30-39) and having a lower socioeconomic status.
Mohd Mustafah et al, 2017^22^	- Assess the frequency of individuals returning to work, along with their sociodemographic traits, medical conditions, and overall quality of life. - Additionally, investigate the elements that influence the likelihood of resuming employment following a cardiac incident.	- There were 74 participants who returned to work and 38 who did not. - The prevalence of return to work was 66.1% [95% CI: 57.2–75.0]. Among those who returned to work, 93.2% returned to the same job and 6.8% switched to a less demanding job. - Factors associated with return to work included age (*P* = 0.049), angiographic status (*P* < 0.001), DM status (*P* = 0.012), and MCS (*P* = 0.022). - Participants with single-vessel disease and double-vessel disease were 8.9 times and 3.8 times more likely to return to work compared to those with triple-vessel disease. - Those without DM were 3.7 times more likely to return to work compared to those with DM. - Increasing age is a negative predictor of return to work [Adj. OR: 0.92 (95% CI: 0.84–0.99)]. A higher MCS score predicted a better chance of return to work [Adj. OR: 1.05, 95% CI: 1.01–1.09)].	Increasing age, involvement of multiple vessels, and the presence of diabetes mellitus (DM) were identified as adverse predictors for returning to work. Conversely, a higher Mental Component Summary (MCS) score was associated with a greater likelihood of returning to work. In this study, psychological well-being emerged as the sole modifiable factor influencing the ability to return to employment.

Legend: MI – Myocardial Infarction; CS – Cardiogenic Shock; METs – Metabolic Equivalents; HADS – Hospital Anxiety and Depression Scale; FEV1 – Forced Expiratory Volume; FVC – Forced Vital Capacity; FEV1/FVC – FEV1/FVC ratio; RTW – Return to Work; DM – Diabetes Mellitus; CABG – Coronary Artery Bypass Graft Surgery; HTN – Hypertension; MCS – Mental Component Summary; STEMI – ST-Elevation Myocardial Infarction; PCI – Percutaneous Coronary Intervention; ACS – Acute Coronary Syndrome.

**Table 4:** Information on Disease Characterization, Length of Hospital Stay, Return to Work, and Comorbidities Included in the Systematic Review (n = 12)

**Author, Year**	**Disease Severity**	**Length of Stay**	**Time to Return**	**Comorbidities**
Sun et al, 2022^11^	- 77.9% of participants had a history of myocardial infarction (MI). - Angioplasty was performed on 112 individuals, accounting for 82.3% of the cohort. - At baseline, 119 subjects, or 87.5%, exhibited a left ventricular ejection fraction (LVEF) of 40% or higher.	No data	3 months after hospital discharge	- Number per patient (34.6%, 32.4%, and 30.1%) one, none, and two, respectively - Previous MI, 77.9% - Hypertension (HTN), 48.50% - Diabetes Mellitus (DM), 30.1%
Stendardo et al, 2018^12^	- LVEF 50.22 ± 8.16	Median length of stay was 4 (IQR 4–6) days	1 and 2 months and 1 year after hospital discharge	- In 32.35% of study participants, MI was associated with other comorbidities, the most prevalent being: HTN (57%), DM (15%), and Depression (7%)
Jiang et al, 2018^13^	- 84 (5.4%) had LVEF < 40% - Killip Class 3-4, 63 (4%) patients - Most patients (1,165 [74.4%]) underwent PCI during hospitalization	Length of stay, median (IQR), 11.0 (8.0-14.0) days	1, 6, and 12 months after hospital discharge	- The five most common comorbid conditions identified in the population were: Hypertension (727 individuals, representing 46.4%), a history of Coronary Artery Disease (620 individuals, or 39.6%), Dyslipidemia (492 individuals, accounting for 31.4%), Heart Failure (345 individuals, which corresponds to 22.0%), and Type 2 Diabetes Mellitus (292 individuals, equating to 18.6%).
Butt et al, 2018^14^	- All patients underwent CABG - 1816 (70%) of participants aged 56-60 years had no hospitalizations in the first year after discharge	No data	5 weeks to 1 year after CABG	- Most participants had a *Charlson Comorbidity Index* of 0–2 - Most prevalent comorbidities: HTN, MI, and DM
Warraich et al, 2018^15^	- LVEF 51.4 (10.7) - Previous MI: 608 (14.4%) - Previous CABG: 206 (4%) - Previous PCI: 672 (15.9%)	Length of stay 2.9 days	1 year after hospital discharge	- The predominant comorbid conditions identified were Heart Failure at 8.9%, Arrhythmia at 7.3%, Diabetes Mellitus at 9.3%, and Depression at 4.6%.
Babić et al, 2015^16^	- Anterior myocardial infarction was observed in 65 patients (44.8%), while inferior myocardial infarction occurred in 70 patients (48.3%). - Culprit arteries identified included the left anterior descending artery (64 cases, 44.2%), the right coronary artery (56 cases, 38.6%), and the circumflex artery (25 cases, 17.2%)	The average length of stay was 11.84 ± 4.62 days	The time to return to work for those who considered their quality of life worse was 60 days, 25th-75th percentile (61.0 – 244), and for those who considered it the same, it was 43 days (61 – 122)	Most prevalent comorbidities: HTN 82 (56.6%), dyslipidemia 79 (54.5%), and DM 24 (16.6%)
Osler et al, 2014^17^	- 8,777 people underwent CAG and 6,257 PCI	No data	30 days, 1 year, 2 years, and 5 years after ACS	4,246 had 1-2 comorbidities
de Jonge et al, 2014^18^	- LVEF < 40% was observed in 12 patients (14.0%) at the 3-month mark and in 19 patients (14.3%) at the 12-month mark, both of whom returned to work (RTW). - For those with a Killip classification of ≥ 2, the rates of RTW were patients (3.5%) at 3 months and 4 patients (3.0%) at 12 months.	No data	At 3 and 12 months after MI	The comorbid conditions observed in patients who resumed employment at 3 and 12 months post-treatment included dyslipidemia, hypertension (HTN), and diabetes mellitus (DM).
Worcester et al, 2014^19^	- The incidence of Killip class 2 or 3 among medical patients was recorded at 22 cases, representing 12.1%. - A left ventricular ejection fraction (LVEF) of less than 45% was observed in 61 patients, accounting for 18.4%. - Among surgical patients, the occurrence of concomitant valve surgery was noted in 31 instances, equating to 15.1%. - Additionally, the use of three or more grafts in surgical patients was found in 127 cases, which constitutes 62.3%.	- Medical patients: Average length of stay is 5.05 days, with a standard deviation of 3.21. - Surgical patients: Average length of stay is 9.92 days, with a standard deviation of 3.40.	Up to 12 months	DM 87 (22.6%) and other comorbidities 45 (11.6%)
Lauridsen et al, 2022^20^	- Majority of patients underwent CAG during hospitalization (CS: 93% vs. without CS: 90%)	4 [2-16] days; without CS 4 [2-14] and with CS 13 [4-94] days	1 year after hospital discharge	- Charlson Comorbidity Index: Frequent: 0 (78%), 1-2 (18%), and > 2 (5%) - HTN (20%), DM (8%), Cancer (3%), Cerebrovascular Disease (3%), Peripheral Vascular Disease (2%)
Smedegaard et al, 2017^21^	- 16,278 (72.7%) underwent CAG, followed by 55.3% PCI	No data	Within 1 month, 3 months, and 1 year after hospital discharge	- The most prevalent comorbidities were Heart Failure (8.9%), Arrhythmia (7.3%), DM (9.3%), and Depression (4.6%)
Mohd Mustafah et al, 2017^22^	- Among those who resumed employment, 36 individuals (48.6%) underwent coronary angiography (CAG) and 11 individuals (14.9%) received coronary artery bypass grafting (CABG). - A total of 61 participants (82.4%) were classified as NYHA class 1. - Participants with single-vessel and double-vessel diseases had a return-to-work likelihood that was 8.9 times and 3.8 times higher, respectively, than that of participants with triple-vessel disease.	No data	No data	- Of the participants who did not return to work, 33 (86.8%) had Dyslipidemia, 27 (71.1%) HTN, and 23 (60.5%) DM.

Legend: MI – Myocardial Infarction; LVEF – Left Ventricular Ejection Fraction; HTN – Hypertension; DM – Diabetes Mellitus; CS – Cardiogenic Shock; CAG – Coronary Angiography; PCI – Percutaneous Coronary Intervention; CABG – Coronary Artery Bypass Grafting; IQR – Interquartile Range; NYHA – New York Heart Association; ACS – Acute Coronary Syndrome.

 Regarding educational level, most participants had an education level below high school, with mentions of vocational education, basic education,^14,21^ and secondary education,^22^ high school,^17^ and low to medium high school levels.^18^ Only five cohort studies provided insights into participants’ job classifications; predominant occupations included office/coordination roles and manual laborers, ^12^ agricultural workers,^13^ private sector employees across specialized categories and business professionals,^22^ as well as government agency staff.^16^ de Jonge et al also noted that most participants who returned to work had sedentary jobs, classified as light work.^18^

 As for being active in the labor market at the time of the MI, among groups of people working or on leave, no more than three articles reported that 41.2%,^11^ 51%,^15^ and 56.9%^21^ of the participants were employed. The prevalence of return to work (RTW) after the acute event varied during the follow-up period. After one month, the return rate was 21.5%,^12^ 37%,^17^ and 41.7%^21^; after two months, it was 59.2%^12^; after three months, it was 46.2%,^18^ 86.4%,^21^ and 87.5%^11^; after four months, 91.1%^19^; after six months, 83.6%^14^; within one year, 55.9%^13^; one year: 76.9%,^18^ 79.9%,^19^ 80%,^14^ 91.1%,^21^ 92.7%^12^ and MI without SC 83% and with SC 52%^20^; after two years, 91.6%.^14^ Only one study presented the return rate without a time limit, with a general rate of 66.1%.^22^

 Among those who returned to work, researchers from Malaysia (2017) identified that 93.2% returned to the same job, while 6.8% switched to a less demanding job.^22^ Osler et al noted that 17.9% of patients reported a significant salary reduction compared to the period before the coronary event.^17^ Another point concerns job termination within one or two years, particularly among those who had received medical leave.^14,17,21^ The average time of work leave was 44 days,^12^ 125.83 ± 125.04 days,^16^ and 8.62 weeks.^19^ The main circumstances leading to not returning to work were the inability to work and/or choosing not to work due to the MI,^13,17^ early retirement/disability retirement,^13,14^ regular retirement,^15,17,19^ unemployment,^13,15,17,19^ dismissal,^15,17^ paid medical leave,^14,17^ disability pension, death, emigration,^14^ and unspecified reasons.^13^

 Another factor that interfered with RTW was the presence of depression^12,15,18,21^ and anxiety,^12,18^ as noted in four articles. Regarding comorbidities, Table 3 presents results on this variable from all articles. The most prevalent comorbidities were systemic arterial hypertension (SAH),^11,12,14-16,18,20,22^ diabetes mellitus (DM),^11,12,14-16,18-22^ dyslipidemia,^13,16,18,22^ and depression.^12,21^ Regarding the severity of the disease, seven articles provided data on the Left Ventricular Ejection Fraction (LVEF), with reduced EF (< 40),^13,18^ borderline EF (41-49), ^11,19^ and preserved EF ( ≥ 50). ^12,15^ Three articles used the *Killip Classification*: Killip 3-4,^16^ ≥ 2, ^18^ and 2-3. ^19^ Regarding the length of hospital stay, six articles provided data, in days, ranging from 2.9,^15^ 4, ^12,20^ 5.05-9.92, ^19^ to 11.^13,16^ The time to return to work was assessed at five weeks, ^14^ one month, ^12,13,17,21^ two months,^12,16^ three months, ^11,18,21^ six months, ^13^ one year, ^12,14,15,17-21^ two years, and five years ^17^ after hospital discharge or after MI.

 Regarding the assessment of the methodological quality of the articles, all the cohort studies were classified as good quality, with Stendardo et al, ^12^ Warraich et al, ^15^ and Worcester et al ^19^ not clearly describing the study population. Additionally, none of the included articles justified the sample size (Table 5).

**Table 5:** Quality Assessment Tool for Observational Cohort and Cross-Sectional Studies (n = 12)

**Study**	**1**	**2**	**3**	**4**	**5**	**6**	**7**	**8**	**9**	**10**	**11**	**12**	**13**	**14**	**Quality**
Sun et al, 2022^11^	Y	Y	Y	Y	N	NA	Y	N	Y	Y	Y	NA	Y	Y	Good
Stendardo et al, 2018^12^	Y	N	Y	NR	N	NA	Y	Y	Y	Y	Y	NA	Y	Y	Good
Jiang et al, 2018^13^	Y	Y	Y	Y	N	NA	Y	Y	Y	Y	Y	NA	Y	Y	Good
Butt et al, 2018^14^	Y	Y	Y	Y	N	NA	Y	Y	Y	Y	Y	NA	Y	Y	Good
Warraich et al, 2018^15^	Y	N	Y	NR	N	NA	Y	Y	Y	N	Y	NA	Y	Y	Good
Babić et al, 2015^16^	Y	Y	Y	Y	N	NA	Y	Y	Y	Y	Y	NA	Y	Y	Good
Osler et al, 2014^17^	Y	Y	Y	Y	N	NA	Y	Y	Y	Y	Y	NA	Y	Y	Good
de Jonge et al, 2014^18^	Y	Y	Y	Y	N	NA	Y	Y	Y	Y	Y	NA	Y	Y	Good
Worcester et al, 2014^19^	Y	N	Y	NR	N	NA	Y	Y	Y	Y	Y	NA	Y	Y	Good
Lauridsen et al, 2022^20^	Y	Y	Y	Y	N	NA	Y	Y	Y	N	Y	NA	Y	Y	Good
Smedegaard et al, 2017^21^	Y	Y	Y	Y	N	NA	Y	Y	Y	Y	Y	NA	Y	Y	Good
Mohd Mustafah et al, 2017^22^	Y	Y	Y	Y	N	NA	Y	Y	Y	NR	Y	NA	Y	Y	Good

1. Was the research question or objective in this paper clearly stated? 2. Was the study population clearly specified and defined? 3. Was the participation rate of eligible persons at least 50%? 4. Were all the subjects selected or recruited from the same or similar populations (including the same time period)? Were inclusion and exclusion criteria for being in the study prespecified and applied uniformly to all participants? 5. Was a sample size justification, power description, or variance and effect estimates provided? 6. For the analyses in this paper, were the exposure(s) of interest measured prior to the outcome(s) being measured? 7. Was the timeframe sufficient so that one could reasonably expect to see an association between exposure and outcome if it existed? 8. For exposures that can vary in amount or level, did the study examine different levels of the exposure as related to the outcome (e.g., categories of exposure, or exposure measured as continuous variable)? 9. Were the exposure measures (independent variables) clearly defined, valid, reliable, and implemented consistently across all study participants? 10. Was the exposure(s) assessed more than once over time? 11. Were the outcome measures (dependent variables) clearly defined, valid, reliable, and implemented consistently across all study participants? 12. Were the outcome assessors blinded to the exposure status of participants? 13. Was loss to follow-up after baseline 20% or less? 14. Were key potential confounding variables measured and adjusted statistically for their impact on the relationship between exposure(s) and outcome(s)? Y, yes; N, no; CD, cannot determine; NA, not applicable; NR, not reported

## Discussion

 The findings from this systematic review indicate that the rates of return-to-work (RTW) for individuals following a myocardial infarction (AMI) fluctuate based on the duration of follow-up, showing an upward trend over time. After one year post-event, the RTW rate is notably high, ranging from approximately 76.9% to 92.7%. ^12,14,18,19,21^ It is crucial to highlight that several factors influence the likelihood of an earlier return to work, including age, educational attainment, income level, type of employment—particularly those involving sedentary tasks or roles focused on office coordination—and self-employment status. ^12,14,18,21^ Consequently, the intricate relationship between myocardial infarction and return-to-work encompasses various physiological, socioeconomic, and sociopsychological dimensions that shape individuals’ perceptions and experiences regarding their capacity to resume regular activities.

###  Severity of the disease

 Regarding the disease’s severity, most participants demonstrated either preserved or borderline left ventricular ejection fraction (LVEF). A lower incidence of LVEF values below 40%, along with a higher Killip class and myocardial infarction (MI) history, correlated with returning to work (RTW) compared to those who did not. ^18^ Mohd Mustafah et al indicated that individuals with single-vessel and two-vessel disease were 8.9 times and 3.8 times more likely, respectively, to return to work than their counterparts with three-vessel disease. ^22^ In contrast, researchers from Croatia found no significant association between traditional risk factors for arterial disease—such as affected myocardial wall segments and culprit coronary arteries—and permanent work cessation or the duration until returning to work. ^16^ Consequently, utilizing clinical factors as variables for assessing RTW following MI remains a contentious issue due to its connection to various influencing elements.

###  Length of hospital stay

 Regarding the duration of hospitalization, one study indicated that there were no notable differences in return-to-work (RTW) rates between patients who resumed employment and those who did not. ^13^ Conversely, another investigation found that hospital stays exceeding 30 days and instances of anoxic brain injury during admission were linked to a diminished probability of returning to work, ^20^ alongside an increase in the number of hospitalization days. ^19^ These results are corroborated by Jiang et al, which emphasizes that complications occurring during a myocardial infarction can prolong the RTW process. ^13^ Consequently, the length of hospitalization reported in this study may be considered brief, likely reflecting improvements in treatment modalities and care protocols for the participants involved.

###  Comorbidity

 All studies incorporated in this review examined various forms of comorbidity. The findings clearly indicated that individuals with a greater number of comorbid conditions had reduced chances of re-entering the workforce. ^14,15,20,21^ Among the most commonly observed comorbidities were diabetes mellitus (DM), systemic arterial hypertension (SAH), and acute myocardial infarction (AMI). Specifically, research conducted in Malaysia revealed that participants without DM were 3.7 times more likely to resume work compared to those diagnosed with the condition. ^22^ The advancements in myocardial infarction treatment, along with the rising number of survivors post-event, underscore the necessity for effective management of disease burden, as inadequate management may result in additional negative consequences.

###  Anxiety and depression

 A significant theme that emerged from the reviewed studies was the connection between the inability to return to work or a diminished likelihood of doing so among individuals exhibiting symptoms of anxiety and depression. de Jonge et al found that the presence of any anxiety disorders within the first three months following a myocardial infarction (MI) correlated with a heightened risk of not resuming work. ^18^ This observation is supported by Warraich et al, who noted that over the course of a year, patients experiencing negative changes in employment were more likely to have a Patient Health Questionnaire-2 (PHQ-2) score exceeding 3, indicating potential depression, as well as reduced EQ-5D VAS scores, reflecting lower quality of life. ^15^ These results underscore a diminished self-assessment of health status when individuals do not return to work, highlighting the role of positive mood as a predictive factor for successful reintegration into the workforce. ^12^

###  Time away from work

 In a prospective cohort study conducted in Italy, the average duration of time off work was found to be 44 days. ^12^ Similarly, another investigation revealed that the typical span from 30 days post-diagnosis to either retirement, death, or cessation of work was approximately 4.1 years. ^17^ Among employees who were unable to return following an acute myocardial infarction (AMI), the reasons for not resuming work varied significantly; these included inability to perform job duties, early retirement, receiving a disability pension, termination of employment, or death. ^13^ Researchers have indicated that the probability of experiencing disability or opting for voluntary early retirement was notably higher among specific groups—namely women, individuals aged over 50 years, those with limited educational backgrounds, people in low-skill occupations, those living with a partner, and individuals who were on sick leave or unemployed 30 days after their hospital admission. Additionally, factors such as having undergone myocardial revascularization and the presence of comorbidities also contributed to this increased likelihood.^17^

 This review underscores critical factors associated with the return to work following a myocardial infarction (MI) and identifies elements that contribute to prolonged absence from employment. The findings presented offer insights that can inform intervention strategies and guide future research aimed at minimizing the duration of time away from work, thereby alleviating costs for both individuals and society at large.

 While a majority of the studies reviewed exhibit strong methodological rigor and provide data on temporal trends, certain limitations are evident concerning the included research: notably, the small sample sizes and specific characteristics of the countries where these outcomes were assessed. Of the twelve studies analyzed, four were conducted in Denmark, with no representation from Latin America or Africa, potentially affecting the observed return-to-work rates. Furthermore, a significant limitation lies in the considerable variability in how results were reported across studies, which precluded the possibility of conducting a meta-analysis.

## Conclusion

 The rate of individuals returning to work following a Myocardial Infarction is notably high within the first year. This phenomenon is influenced by various physical, psychological, and social factors, alongside the identification of mechanisms that facilitate an expedited return to employment. Nevertheless, further research is essential, particularly studies conducted across diverse populations and separating professional categories, to gather more thorough insights.

## Competing Interests

 No conflicts of interest.

## Ethical Approval

 Not applicable.
